# Automated Grading of Cerebral Vasospasm to Standardize Computed Tomography Angiography Examinations After Subarachnoid Hemorrhage

**DOI:** 10.3389/fneur.2020.00013

**Published:** 2020-01-30

**Authors:** Axel Neulen, Svenja Kunzelmann, Michael Kosterhon, Tobias Pantel, Maximilian Stein, Manfred Berres, Florian Ringel, Marc A. Brockmann, Carolin Brockmann, Sven R. Kantelhardt

**Affiliations:** ^1^Department of Neurosurgery, University Medical Center of the Johannes Gutenberg-University of Mainz, Mainz, Germany; ^2^Department of Mathematics and Technology, University of Applied Sciences Koblenz, Remagen, Germany; ^3^Department of Neuroradiology, University Medical Center of the Johannes Gutenberg-University of Mainz, Mainz, Germany

**Keywords:** computed tomography angiography, computed tomography perfusion imaging, grading, vasospasm, subarachnoid hemorrhage, delayed cerebral ischemia

## Abstract

**Background:** Computed tomography angiography (CTA) is frequently used with computed tomography perfusion imaging (CTP) to evaluate whether endovascular vasospasm treatment is indicated for subarachnoid hemorrhage patients with delayed cerebral ischemia. However, objective parameters for CTA evaluation are lacking. In this study, we used an automated, investigator-independent, digital method to detect vasospasm, and we evaluated whether the method could predict the need for subsequent endovascular vasospasm treatment.

**Methods:** We retrospectively reviewed the charts and analyzed imaging data for 40 consecutive patients with subarachnoid hemorrhages. The cerebrovascular trees were digitally reconstructed from CTA data, and vessel volume and the length of the arteries of the circle of Willis and their peripheral branches were determined. Receiver operating characteristic curve analysis based on a comparison with digital subtraction angiographies was used to determine volumetric thresholds that indicated severe vasospasm for each vessel segment.

**Results:** The automated threshold-based volumetric evaluation of CTA data was able to detect severe vasospasm with high sensitivity and negative predictive value for predicting cerebral hypoperfusion on CTP, although the specificity and positive predictive value were low. Combining the automated detection of vasospasm on CTA and cerebral hypoperfusion on CTP was superior to CTP or CTA alone in predicting endovascular vasospasm treatment within 24 h after the examination.

**Conclusions:** This digital volumetric analysis of the cerebrovascular tree allowed the objective, investigator-independent detection and quantification of vasospasms. This method could be used to standardize diagnostics and the selection of subarachnoid hemorrhage patients with delayed cerebral ischemia for endovascular diagnostics and possible interventions.

## Introduction

Spontaneous subarachnoid hemorrhage (SAH) is a subtype of hemorrhagic stroke (1–3). In most cases, SAH is caused by the rupture of an intracranial aneurysm, with the bleeding resulting in a considerable increase in intracranial pressure. The resulting decline in cerebral perfusion pressure then induces transient global cerebral ischemia. Early brain injury can occur as an immediate effect of the bleeding and the transient global cerebral ischemia (4, 5). The intracranial pressure eventually decreases and cerebral perfusion returns (5). Of the patients who survive the initial bleeding event, up to 30% then suffer delayed cerebral ischemia (DCI) during the first weeks after SAH (4, 5). DCI is defined as a new global or focal neurological impairment that lasts for at least 1 h or a new cerebral infarction that was not immediately apparent after aneurysm occlusion and which is attributable to ischemia and not to other causes (6). The underlying pathophysiology of DCI is only partially understood. However, it is accepted that its genesis is multifactorial, with factors including vasospasms of the large intracranial arteries, disturbance of the microcirculation by vasospasms and the microthrombosis of microvessels, cortical spreading depression, and inflammatory effects (4–7).

Endovascular interventions can be applied as rescue therapies when patients experience severe vasospasms of the large intracranial arteries that threaten to impair cerebral perfusion; possible interventions include angioplasty of the spastic segments or the intra-arterial administration of vasodilating drugs (6, 8–12). The gold standard for the diagnosis of severe vasospasm with an indication for endovascular intervention is digital subtraction angiography (DSA), which allows an assessment of the severity of the vasospasm and of its effects on the perfusion of subsequent vascular territories (6, 8–10). However, DSA is an invasive and technically demanding intervention and so is not suitable as a screening tool. The indication for DSA is therefore commonly based on a combination of non- or less-invasive techniques, including a clinical examination (if the patient is conscious), a transcranial Doppler ultrasound examination of cerebral blood flow velocities, cerebral perfusion computed tomography (CTP), and cerebral computed tomography angiography (CTA) (6, 8–10).

Studies have shown that CTP can be used to detect cerebral hypoperfusion and predict cerebral infarction (13–18). A prospective observational study, however, reported that CTP had a positive predictive value (PPV) of only 29% for forecasting severe vasospasm or new cerebral infarction within the subsequent 72 h (19). Other studies have shown that CTA can detect cerebral vasospasms with a sensitivity and specificity comparable to that of DSA (20–22). Therefore, CTA is commonly regarded as offering a valuable adjunct to CTP for deciding whether DSA and endovascular intervention are indicated. However, although several studies have established objective scores and defined thresholds for the evaluation of CTP (13–19), there remains a lack of objective parameters or thresholds for the interpretation of CTA.

One possible approach to standardizing the assessment of CTA and exploiting additional diagnostic information is to perform digital analyses of cross-sectional imaging data of the cerebrovascular tree. This could potentially detect the extent of the angiographic vasospasm and grade it automatically in an investigator-independent manner. Our group has developed a method that enables a highly accurate virtual reconstruction of the intracranial vascular tree from CTA data and a volumetric analysis of entire vessel segments (23). In a previous study, we performed a volumetric evaluation of the M1 segment of the middle cerebral artery based on CTA data acquired as part of the DCI diagnostics for a cohort of SAH patients (24). We determined a volumetric threshold that could predict with moderate sensitivity and fair specificity the patients for whom endovascular treatment would subsequently be indicated (according to the diagnostic standards of our hospital) or who would experience a new cerebral infarction within 72 h. This demonstrated that such an assessment can identify severe vasospasms. However, the study was limited by focusing exclusively on the M1 segment.

In the present study, we extended the approach to an analysis of all the vessel segments of the circle of Willis and their major peripheral branches. This study had two aims: to determine volumetric thresholds for each vessel segment as an objective measure for diagnosing severe vasospasm; and to test whether the automated detection of severe vasospasm by volumetric CTA evaluation, alone or in combination with CTP, could be used to predict the subsequent indication for endovascular vasospasm treatment in this study cohort.

## Materials and Methods

### Patients and Study Design

**Figure 1:**
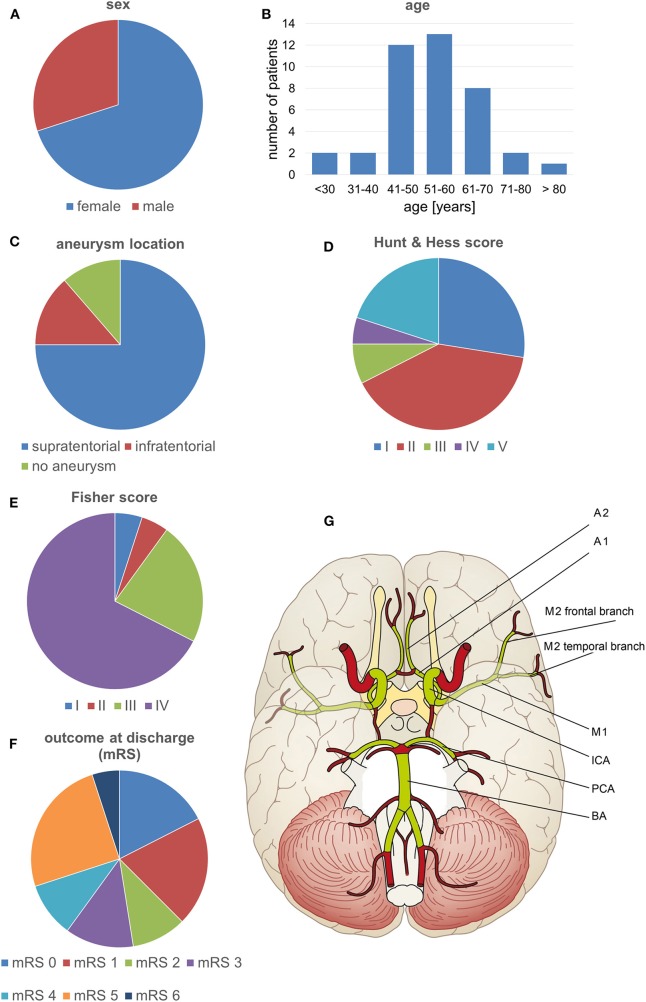
Patient characteristics and the vessel segments examined. **(A–F)** Characteristics of the patients included in the study. **(G)** The volumetrically evaluated vessel segments.

We performed a retrospective chart review and analysis of imaging data from 40 consecutive SAH patients admitted to our institution between November 2013 and December 2015 and who underwent surveillance for vasospasm. These patients were primarily enrolled in an unrelated prospective clinical study of a diagnostic medical device conducted at our institution (registered with ClinicalTrials.gov, identifier NCT02071875; the study is yet to report). Characteristics of the patients are summarized in Figures 1A–F.

All the procedures in this study were conducted in accordance with the ethical standards of our institutional review board (Ethikkommission der Landesaerztekammer Rheinland Pfalz), which approved the study protocol, and with the principles of the 1964 Helsinki Declaration and its later amendments. Informed consent was obtained from all the patients included in this study or their legally authorized representative. The retrospective chart review was also approved by our institutional review board.

### Clinical Management

The SAH patients were managed clinically according to clinical standard protocols described previously (24–26). In brief, the SAH was diagnosed by cranial CT, followed by CTA and DSA to detect aneurysms. After reaching an interdisciplinary consensus, an aneurysm was treated either by surgical clipping or by endovascular coil embolization with or without remodeling. If indicated, cerebrospinal fluid drains were placed to treat hydrocephalus. Multimodal neuromonitoring of intracranial pressure and brain tissue oxygenation (ptiO_2_) was used for sedated patients for whom a detailed neurological examination was not feasible. All patients received oral nimodipine for 21 days after the SAH. DCI was suspected when there was focal or global neurological deterioration, a reduction in ptiO_2_ to <15 mmHg, or increased Doppler sonographic blood flow velocities; blood flow velocities of <120 cm/s were considered non-suspicious for vasospasm, whereas those of >200 cm/s in at least one intracranial vessel segment triggered further diagnostics. For blood flow velocities that were between 120 and 200 cm/s, further diagnostics were initiated upon the discretion of the responsible neurointensivist, taking the course of blood flow velocities in serial examinations into account. CTA and CTP were performed with cases of suspected DCI. Cases of cerebral hypoperfusion that were confirmed on CTP were treated by induced hypertension aiming at a mean arterial pressure of >110 mmHg or a cerebral perfusion pressure of >100 mmHg. DSA was performed only in refractory cases (i.e., no improvement of neurological deficit or no improvement in ptiO_2_ in the case of sedated patients), to perform endovascular treatment (i.e., angioplasty and/or intra-arterial vasospasmolysis with nimodipine). Only these DSAs were evaluated in this study.

### Data Collection

Data were extracted from charts on the patient's sex, age, Hunt and Hess (27) and Fisher (28) grades at hospital admission, outcome at discharge, and localization of the symptomatic aneurysm. CTA, CTP, and DSA imaging data were obtained from the clinical imaging archive. All the data were collected in anonymized files.

### Imaging

CTA and CTP were performed according to standard diagnostic protocols (80 kV; 50 mAs; 40 mL Imeron 400 administered via a power injector at an injection rate of 5 mL/s) using a Toshiba Aquilion 32 CT scanner with 32 slices (512 × 512 pixels; Software Base V3.20°R003//Appl.: V3.10GR004), as described previously (24). Intracranial CTA was recorded from the foramen magnum up to the superior sagittal sinus. The start of acquisition was triggered manually at the moment of maximal contrast in the aortic arch. Images were reconstructed in the axial, sagittal, and coronal planes. The duration of the CTP scan was 45 s; four brain segments were scanned with 30 layers in each, resulting in 120 images.

Catheter angiography was performed using standard intraarterial biplane DSA (Biplane Allura Xper FD System, Philips, The Netherlands). After placement of the femoral sheath in the common femoral artery, cerebral DSA was performed, including the circulation along the left and right internal carotid arteries as well as in at least one vertebral artery. The DSA of the cerebral vessels was documented in the standard projection and oblique projections.

The CTA, CTP, and DSA images were reviewed using Sectra Workstation IDS7 Software Version 18.2.8.3923 (Sectra AB, Linköping, Sweden).

### Volumetric Analysis of CTA Data

**Figure 2:**
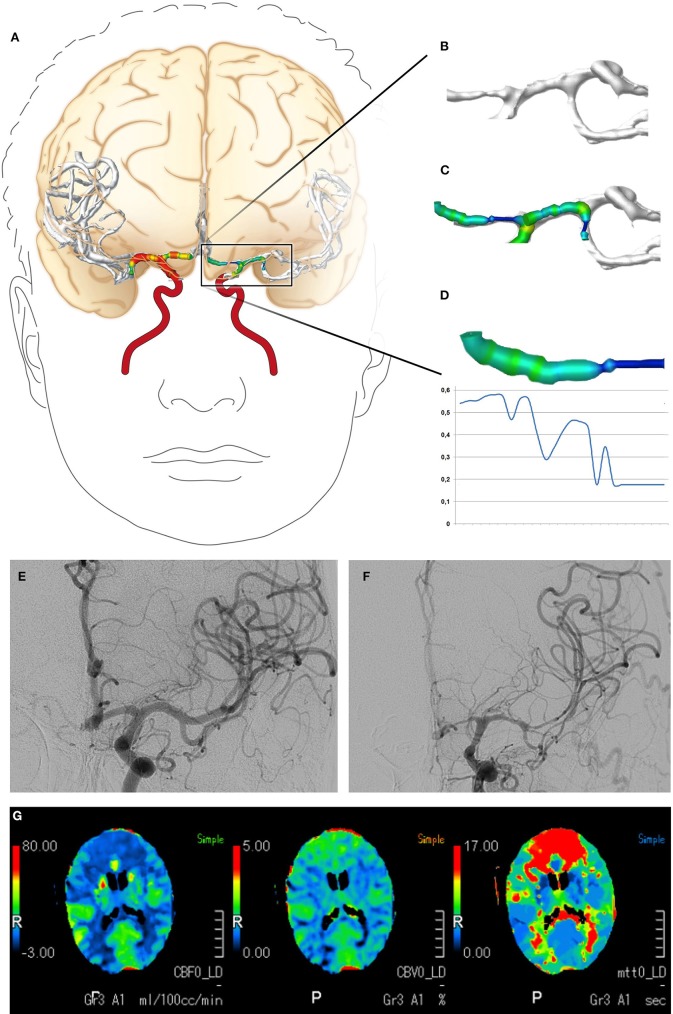
Digital reconstruction and volumetric analysis of the cerebrovascular tree from CTA data, the classification of vasospasm from DSA data, and the evaluation of CTP. **(A–D)** The volumetric evaluation of CTA data. The cerebrovascular tree was virtually reconstructed and a volumetric analysis of the defined vessel segments was performed. **(E,F)** Examples of vasospasm scoring. The vessel diameters of the A1 and M1 segments declined by >50%, resulting in vasospasm scores of 2. **(G)** Example of CTP with cerebral hypoperfusion (elongated mean transit time, reduced cerebral blood flow) in both anterior cerebral artery territories. CTA, computed tomography angiography; DSA, digital subtraction angiography; CTP, computed tomography perfusion imaging.

The CTA data sets acquired within the scope of the diagnostics for DCI were subjected to a volumetric analysis of the intracranial vessels using Amira software version 5.4.2 (FEI Visualization Sciences Group, Hillsboro, OR, USA) following a standardized protocol (23, 24). In brief, the bone was virtually subtracted and the intracranial vascular tree was then virtually reconstructed by applying a standardized windowing procedure (23, 24) shown to have high interrater agreement in results for vessel volumes (24). The vessel volume and the length of the corresponding vessel segment were calculated according to a standardized algorithm, as previously described (23, 24, 29, 30), for the following vessel segments: the basilar artery, the posterior cerebral artery, the internal carotid artery, the M1 and M2 segments of the middle cerebral artery, and the A1 and A2 segments of the anterior cerebral artery. To account for individual differences in the length of the vessel segments, the vessel volumes were expressed in relation to the vessel length (volume/length ratio). The volume/length ratios of the PCA were calculated considering P1 and P2 segments together; in case of an atretic P1 segment with fetal PCA (a single case), the PCA volume/length ratio was calculated by only considering the P2 segment. Figures 1G, 2 provide a detailed overview of the vessel segments analyzed and illustrate the volumetric analysis of a vessel segment.

### Grading of Vasospasm

Vasospasm was diagnosed and graded for the present study by an experienced neuroradiologist using the DSA data acquired immediately after CTA and prior to any endovascular vasospasm treatment. The neuroradiologist also had access to the DSA performed at hospital admission to search for the bleeding source, and to the corresponding CTA. She was blinded to all other examinations. The neuroradiologist identified the vessel segments of the circle of Willis and their major branches. Each vessel segment was then graded according to a grading system for vasospasms (13), as follows: 0, no vasospasm; 1, vasospasm with <50% change in the vessel diameter compared to the initial DSA; or 2, vasospasm with >50% narrowing of the vessel diameter compared to the initial DSA. Vasospasms graded 2 were considered severe. An example is shown in Figure 2.

### Evaluation of CTP

CTP data acquired within the scope of the diagnostics for DCI were evaluated for perfusion deficits using a visual grading score developed by Wintermark et al. (15). The evaluation was performed for the present study by an experienced neuroradiologist blinded to the results of the volumetric evaluation of CTA data. In brief, the neuroradiologist reviewed the mean transit time and cerebral blood flow maps and graded defined anatomical regions (frontal, temporal, parietal, and occipital regions and the thalamus, basal ganglia, and insula) for abnormality as follows: 0, the perfusion appeared normal; 1, perfusion appeared abnormal. A grading other than 0 in at least one of the analyzed regions was considered to indicate cerebral hypoperfusion. An example is shown in Figure 2.

### Statistical Analysis

We used R software version 3.4.1 (https://www.r-project.org/) and the R packages “pROC” and “psych” for the statistical evaluation.

#### Vessel Segment Volume Thresholds for Severe Vasospasm

A threshold value that predicted severe vasospasm (i.e., vasospasm classified as 2) was established for each of the vessel segments. The classifications of vasospasm in single vessel segments on DSA were taken as the gold standard and used in receiver operating characteristic (ROC) curve analyses of the volumetric evaluations from the corresponding CTAs. The cutoff values that maximized the sum of sensitivity and specificity for each segment were identified (31). A bootstrap algorithm was then applied to calculate the optimal threshold and its 95% confidence interval based on 2,000 stratified bootstrap replicates for each single vessel segment. Data for the left and right vessel segments were analyzed together.

#### Diagnostic Value for Predicting Cerebral Hypoperfusion

The volumetric CTA evaluation method was assessed for its diagnostic value in predicting cerebral hypoperfusion, with the presence of hypoperfusion on CTP used as the gold standard. All CTA examinations performed together with CTP were used in the analysis and the vessel segment thresholds (described in the previous section) were applied to the vessel volumes. Three different criteria were considered: a minimum of one vessel segment was below the threshold on CTA; a minimum of two vessel segments were below the threshold; or a minimum of three vessel segments were below the threshold. Contingency tables were created and the sensitivity, specificity, and positive and negative predictive values (PPV, NPV) were calculated.

#### Diagnostic Value for Predicting Subsequent Endovascular Vasospasm Therapy

The volumetric CTA evaluation, CTP, and the combination of CTA and CTP were assessed for their diagnostic value for predicting subsequent endovascular vasospasm therapy. The aim was to assess whether volumetric CTA evaluation or CTP alone, or the combination of both, could predict the presence of vasospasm with impeding infarction if no endovascular treatment is performed. The gold standard used as an indication of the presence of severe vasospasm and impeding infarction was that the patient underwent endovascular vasospasm treatment (indicated according to the clinical standard of the hospital) within 24 h of the CTA examination.

CTA was considered pathological if a minimum of one, two, or three vessel segments were found to be below the respective threshold. The evaluation of CTP for perfusion deficits was considered pathological if it yielded a score of at least 1 point (as described in section Evaluation of CTP). The combination of CTP and CTA was considered pathological when both these conditions were true. For each of these assessments, contingency tables were created and the sensitivity, specificity, PPV, and NPV were calculated.

## Results

### Diagnosis of DCI With CTA, CTP, and DSA

A total of 110 CTA examinations for suspected DCI performed during hospitalization were identified. Of these, nine CTA data sets could not be analyzed digitally for technical reasons. Finally, 101 CTA data sets (for 33 patients) were subjected to the volumetric evaluation.

Subsequent DSA for interventional vasospasm treatment was available for 20 of the volumetrically evaluated CTA data sets (for 10 patients). These 20 sets of CTA examinations and corresponding DSAs were used to determine the thresholds for severe vasospasm.

CTP examinations with corresponding cerebral blood flow and mean transit time maps were available for 96 of the volumetrically evaluated CTA data sets (for 33 patients). These 96 sets of CTA examinations with corresponding CTP were used to compare the volumetric CTA and CTP evaluations and to determine their diagnostic value for predicting subsequent endovascular vasospasm treatment. Within these, 19 examinations (for 10 patients) had been followed by DSAs for endovascular vasospasm treatment within 24 h.

A flow chart summarizing patient and imaging data is shown in the Supplementary Material.

### Vessel Segment Volume Thresholds for Severe Vasospasm

The 20 DSA data sets included 63 severe (i.e., grade 2) vasospasms, located as follows: internal carotid artery, 8; M1 segment, 10; M2 segment frontal branch, 7; M2 segment temporal branch, 12; A1 segment, 5; A2 segment, 11; posterior cerebral artery, 5; and basilar artery, 5. We did not calculate a specific threshold for the vertebral artery because of the marked variability of individual diameters between sides and individuals (32). The results of the ROC curve analyses to determine a threshold for each of the defined vessel segments that indicated severe vasospasm are presented in Table 1.

**Table 1:** Volumetric thresholds that indicate severe vasospasm.

**Vessel segment**	**Threshold (μL/mm)**	**95% CI (μL/mm)**	No. of examinations/patients^*^
BA	4.3	[2.8, 10.3]	18/10
PCA	1.0	[0.9, 2.7]	18/10
ICA	9.2	[5.5, 9.8]	19/10
M1	5.0	[4.6, 8.0]	20/10
M2 (frontal branch)	1.8	[1.0, 4.3]	17/9
M2 (temporal branch)	3.1	[2.2, 3.9]	20/10
A1	2.7	[1.6, 3.7]	19/10
A2	2.1	[1.6, 4.0]	17/10

* *The thresholds were determined using the data for 20 digital subtraction angiography studies (DSAs) from 10 patients. An evaluation was not possible for every segment in every DSA, and the fourth column shows the actual numbers of examinations and patients from which the thresholds were calculated. CI, confidence interval; BA, basilar artery; PCA, posterior cerebral artery; ICA, internal carotid artery*.

### Diagnostic Value for Predicting Cerebral Hypoperfusion

The 96 CTAs for which a corresponding CTP was available included 28 CTPs graded with a score of at least 1, indicating cerebral hypoperfusion. The corresponding CTAs were subjected to the volumetric evaluation and diagnostic values were calculated for the three proposed criteria in which at least one, two, or three segmental vessel volumes were lower than the respective thresholds (considered to indicate a pathologic CTA), with the CTP evaluation considered to be the gold standard. The most successful criterion for predicting cerebral hypoperfusion on the corresponding CTP was that at least one vessel segment had a volume lower than the respective threshold (sensitivity 89%, specificity 41%, NPV 90%, and PPV 39%). The results are shown in Table 2A.

**Table 2A:** Diagnostic value of the volumetric CTA evaluation for diagnosing cerebral hypoperfusion.

**CTP showing hypoperfusion**	**Vasospasm on CTA**		
	**≥1 segment**	**≥2 segments**	**≥3 segments**
N	96	96	96
FP	40	23	9
FN	3	8	11
TP	25	20	17
TN	28	45	59
Sensitivity	89%	71%	61%
Specificity	41%	66%	87%
NPV	90%	85%	84%
PPV	39%	47%	65%

*CTA, computed tomography angiography; CTP, computed tomography perfusion imaging; N, number; FP, false positive; FN, false negative; TP, true positive; TN, true negative; NPV, negative predictive value; PPV, positive predictive value*.

### Diagnostic Value for Predicting Subsequent Endovascular Vasospasm Therapy

For the 96 CTAs with corresponding CTP, 19 were associated with patients who underwent endovascular vasospasm treatment within the 24 h following the scans. The combination of CTA and CTP was superior to either CTA or CTP alone for predicting an impeding infarction requiring endovascular intervention. A CTP showing hypoperfusion in combination with automated CTA evaluation that detected three or more vasospasms yielded a sensitivity for predicting an impeding infarction requiring endovascular intervention of 58%, with specificity 92%, NPV 90%, and PPV 65%. The results are shown in Tables 2B,C.

**Table 2B:** Diagnostic values of CTP and volumetric CTA evaluation for predicting subsequent endovascular vasospasm treatment.

**Endovascular vasospasm treatment**	**CTP**	**Vasospasm on CTA**		
		**≥1 segment**	**≥2 segments**	**≥3 segments**
N	96	96	96	96
FP	16	48	27	13
FN	7	2	3	6
TP	12	17	16	13
TN	61	29	50	64
Sensitivity	63%	90%	84%	69%
Specificity	79%	38%	65%	83%
NPV	90%	94%	94%	91%
PPV	43%	26%	37%	50%

*CTP, computed tomography perfusion imaging; CTA, computed tomography angiography; N, number; FP, false positive; FN, false negative; TP, true positive; TN, true negative; NPV, negative predictive value; PPV, positive predictive value*.

**Table 2C:** Diagnostic value of the combination of CTP and volumetric CTA evaluation for predicting subsequent endovascular vasospasm treatment.

**Endovascular vasospasm treatment**	**CTP & CTA (1 segment)**	**CTP & CTA (2 segments)**	**CTP & CTA (3 segments)**
N	96	96	96
FP	13	8	6
FN	7	7	8
TP	12	12	11
TN	64	69	71
Sensitivity	63%	63%	58%
Specificity	83%	90%	92%
NPV	90%	91%	90%
PPV	48%	60%	65%

*CTP, computed tomography perfusion imaging; CTA, computed tomography angiography; N, number; FP, false positive; FN, false negative; TP, true positive; TN, true negative; NPV, negative predictive value; PPV, positive predictive value*.

## Discussion

In this study, we retrospectively analyzed CTA data acquired as part of the diagnostics for DCI for a cohort of consecutive SAH patients. We digitally reconstructed the cerebrovascular tree and performed a volumetric analysis of the arteries of the circle of Willis and their major branches. By comparison with DSA, we determined volumetric thresholds for each vessel segment that indicated severe vasospasm. This resulted in three main findings: Firstly, the volumetric evaluation of CTA data allowed automated, investigator-independent detection of severe vasospasms. Secondly, identifying CTA vasospasm in at least one vessel segment exhibited a high sensitivity and NPV for predicting the presence of cerebral hypoperfusion on CTP, although the specificity and PPV were rather low. Thirdly, the combination of the automated detection of vasospasm on CTA and cerebral hypoperfusion on CTP was superior to assessments by either CTP or CTA alone for identifying patients with impeding cerebral infarction who underwent interventional DSA for vasospasm treatment to prevent this within 24 h.

It remains difficult to identify the patients who would benefit from endovascular treatment of vasospasm. CTP has been shown to have good sensitivity and specificity for predicting angiographic vasospasm or cerebral infarction in SAH patients during the course of the disease (13–18); however, a prospective study reported that CTP had a PPV of only 29% for predicting severe vasospasm or a new cerebral infarction within the subsequent 72 h (19). Another study reported that a combination of vasospasm identified by CTA together with an increased mean transit time on CTP was the most accurate method for predicting vasospasm as identified by DSA (20). Therefore, in many clinical centers (including ours), the decision on whether or not to perform endovascular vasospasm treatment is based on the clinical symptoms, combined with CTP to detect cerebral hypoperfusion and CTA to estimate the extent of angiographic vasospasm. However, while the CTP evaluation is based on rather objective parameters and scores (13–19), there is a lack of corresponding objective parameters for the evaluation of CTA, resulting in a considerable degree of subjectivity and investigator dependence.

Digital, volumetric, segmental evaluations of the cerebrovascular tree can help to detect and quantify vasospasm in an objective, investigator-independent manner and to exploit more fully the information from the imaging data. However, the finding of the present study that the automated detection of vasospasms on CTA yielded better results when combined with CTP data indicates the need for integrating this technique in a diagnostic workflow that involves CTP, CTA, and all other available sources of relevant information (such as clinical examinations). This could reduce subjective, investigator-dependent influences on the identification of SAH patients who require endovascular therapies. Reducing subjectivity is also a prerequisite for the design of prospective clinical studies for investigating the effectiveness of endovascular vasospasm therapies.

In a notable number of cases, severe vasospasms of at least one vessel segment were detected without hypoperfusion on CTP. We also observed cerebral hypoperfusion in the absence of severe vasospasm in some cases. These observations were consistent with previous studies, which showed that vasospasm and cerebral hypoperfusion can occur independently (30, 33, 34) and that DCI has a multifactorial genesis (4). Again, this highlights the need for multimodal monitoring of SAH patients.

This study had several limitations. It involved a retrospective analysis of the data of a cohort of 40 SAH patients. Although the evaluation confirmed that the digital volumetric assessment of CTA data was technically feasible and could detect vasospasms objectively, these findings reflect the diagnostic and therapeutic processes of our center and may not be directly transferrable to other centers. The design of this study did not take dependence of the data into account. The estimation errors of the diagnostic values are therefore larger than would be estimated from the numbers of examinations. Similarly, confidence intervals for the thresholds are larger than reported in Table 1. For these reasons, a prospective clinical study is needed to examine the suitability of the method for clinical application. The volumetric thresholds determined in this study were not adjusted for individual differences; however, a recent imaging study reported that vessel diameters depend on age and sex (35). Differences associated with sex and age are small compared to those induced by severe vasospasm; nevertheless, a study with a larger patient cohort might reveal significantly different thresholds for severe vasospasm for male and female patients or for different age groups. Finally, differences in the administration of the contrast medium, such as timing and dose, or in the CT scanning protocols and image processing can influence the volumetric values and defined thresholds in the same way as occurs with a non-standardized windowing procedure. We expect these factors to have only a low impact on the results of this study because all the CTA scans investigated were acquired and evaluated in a single center according to highly standardized protocols; nevertheless, this may compromise the transferability of our results (especially the threshold values) to other centers.

## Data Availability Statement

The datasets generated for this study are available on request to the corresponding author.

## Ethics Statement

The studies involving human participants were reviewed and approved by Ethikkommission der Landesaerztekammer Rheinland Pfalz. The patients/participants provided their written informed consent to participate in this study.

## Author Contributions

AN, CB, and SRK: study concept and design. AN, SK, TP, MK, MS, and MB: acquisition of data. AN, SK, CB, and SRK: drafting of the manuscript. AN, SK, TP, MK, MS, MB, FR, MAB, CB, and SRK: critical revision of the manuscript for important intellectual content and analysis and interpretation of data.

### Conflict of Interest

The authors declare that the research was conducted in the absence of any commercial or financial relationships that could be construed as a potential conflict of interest.
