# Supplementary figures and images for: Automated Grading of Cerebral Vasospasm to Standardize Computed Tomography Angiography Examinations After Subarachnoid Hemorrhage
Source: Front Neurol. 2020 Jan 30;11:13. doi: 10.3389/fneur.2020.00013 (PMC7002561; doi:10.3389/fneur.2020.00013)

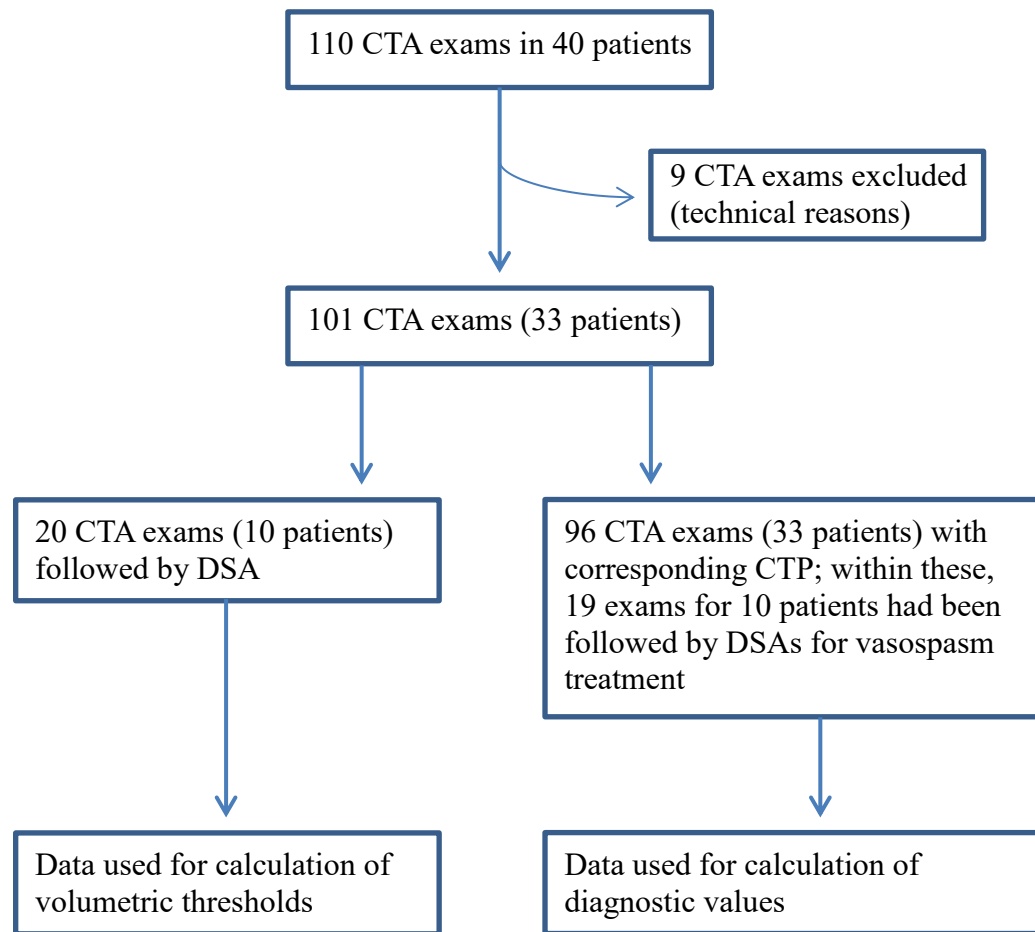

**Supplementary Figure 1.**

Patients and imaging data.

Supplement: Supplementary file 1 [file Data_Sheet_1.PDF]
